# The characteristics and prognosis of different disease patterns of multiple primary lung cancers categorized according to the 8th edition lung cancer staging system

**DOI:** 10.1186/s13019-024-02652-8

**Published:** 2024-04-10

**Authors:** Yalong Wang, Lingling Fang, Xiao Hu, Hongliang Wu, Lina Zhou, Qi Xue, Shugeng Gao, Jie He

**Affiliations:** 1 Department of Anesthesiology, National Cancer Center/National Clinical Research Center for Cancer/Cancer Hospital, Chinese Academy of Medical Sciences and Peking Union Medical College, Beijing, China; 2 Department of Thoracic Surgery, National Cancer Center/National Clinical Research Center for Cancer/Cancer Hospital, Chinese Academy of Medical Sciences and Peking Union Medical College, Beijing, China; 3 Department of Diagnostic Radiology, National Cancer Center/National Clinical Research Center for Cancer/Cancer Hospital, Chinese Academy of Medical Sciences and Peking Union Medical College, Beijing, China; 4 Key Laboratory of Carcinogenesis and Translational Research (Ministry of Education), Department of Pathology, Peking University Cancer Hospital & Institute, Beijing, China

**Keywords:** Multiple primary lung cancer, Prognosis, Lung cancer staging system

## Abstract

**Introduction:**

The 8th edition lung cancer staging system was the first to describe the detailed diagnosis and staging of multiple primary lung cancers (MPLC). However, the characteristics and prognosis of MPLC categorized according to the new system have not been evaluated.

**Method:**

We retrospectively analyzed data from surgically treated MPLC patients in a single center from 2011 to 2013 and explored the characteristics and outcomes of different MPLC disease patterns.

**Results:**

In total, 202 surgically treated MPLC patients were identified and classified into different groups according to disease categories and diagnostic time (multifocal ground glass/lepidic (GG/L) nodules: *n* = 139, second primary lung cancer (SPLC): *n* = 63, simultaneous MPLC (sMPLC): *n* = 171, and metachronous MPLC (mMPLC): *n* = 31). There were significant differences in clinical characteristics between SPLC and GG/L nodule patients and simultaneous and metachronous MPLC patients. The overall 1-, 3-, and 5-year lung cancer-specific survival rates of MPLC were 97.98%, 90.18%, and 82.81%, respectively. Five-year survival was better in patients with multiple GG/L nodules than in those with SPLC (87.94% vs. 71.29%, *P* < 0.05). Sex was an independent prognostic factor for sMPLC (5-year survival, female vs. male, 88.0% vs. 69.5%, *P* < 0.05), and in multiple tumors, the highest tumor stage was an independent prognostic factor for all categories of MPLC.

**Conclusions:**

The different disease patterns of MPLC have significantly different characteristics and prognoses. Clinicians should place treatment emphasis on the tumor with the highest stage as it is the main contributor to the prognosis of all categories of MPLC patients.

**Supplementary Information:**

The online version contains supplementary material available at 10.1186/s13019-024-02652-8.

## Introduction

Lung cancer is the most commonly diagnosed cancer and the leading cause of cancer death worldwide [1]. Multiple primary lung cancer (MPLC), first introduced by Beyreunther in 1924, is a special type of lung cancer in which a single patient presents with at least two primary lung cancers [2]. The cohort of patients diagnosed with MPLC has been growing because of the use of high-resolution chest imaging systems and lung cancer screening programs [3]. For a long time, the accurate diagnosis of MPLC has been a dilemma in clinical practice due to the lack of accurate diagnostic criteria. The previously used clinical diagnostic criteria were proposed by Martini and Melamed in 1975 [4]. Although that standard has a strong clinical practical value, it is imprecise and cannot reflect the true relationship between multiple lung cancers. Finley et al. proposed that the relationship between multiple lung lesions could be judged according to the pathological subtypes of multifocal tumors; others have reported that gene analysis of tumor cells can be used to identify MPLC and lung cancer metastasis [5–8]. However, these methods have drawbacks related not only to histopathological analysis but also to gene analysis. Although the main histopathologic subtypes and gene variations of multiple metastases are generally the same as those of the primary tumor, some independent tumors also show similar histopathological or genetic characteristics. The heterogeneity of homologous tumors and the homogeneity among different tumors affects the interpretation of the relationships among different lung tumors. With regard for the recognition of MPLC, the reality is that we are working blind and have been trying to characterize an entity that we are not (yet) able to observe in its entirety [9].

Due to the lack of a unified diagnosis and staging standard for multiple lung cancers, the diagnostic criteria used in published articles are not consistent. To make the diagnostic and staging criteria of MPLC clearer, the IASLC organized experts to conduct a detailed analysis, and in the 8th edition of the lung cancer staging system released in 2017, multifocus lung cancers were described in detail as a single entity for the first time [10]. In the new version of the staging system, multifocus lung cancer is divided into four distinct disease entities according to patients’ clinicopathological characteristics: second primary lung cancer (SPLC), multiple ground glass/lepidic (GG/L) nodules, separate tumor nodules, and diffuse pneumonic-type lung cancer. Among these, the first two categories are MPLC, and the latter two are lung cancer with pulmonary metastasis [10]. MPLC can also be further divided into simultaneous MPLC (sMPLC) and metachronous MPLC (mMPLC) according to the time of occurrence of multiple lesions. Although MPLC is classified into different categories in the new staging system, to date, there has been no comprehensive comparative analysis of the clinicopathological characteristics, prognosis and prognostic factors of the different MPLCs diagnosed according to the 8th edition staging system. The current retrospective study was conducted to fill this gap.

## Patients and methods

### Patients

**Fig. 1:**
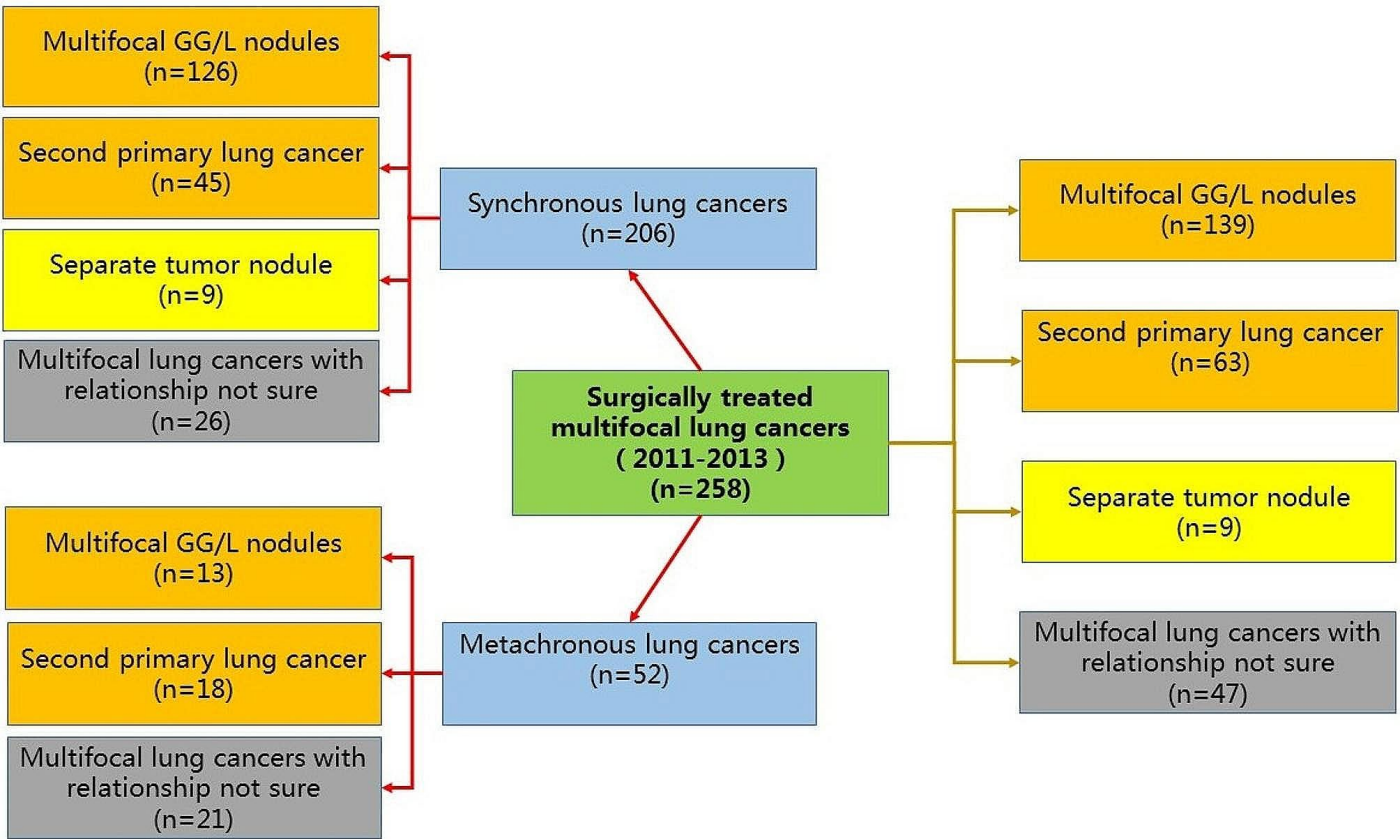
Flow chart of patient selection

**Fig. 2:**
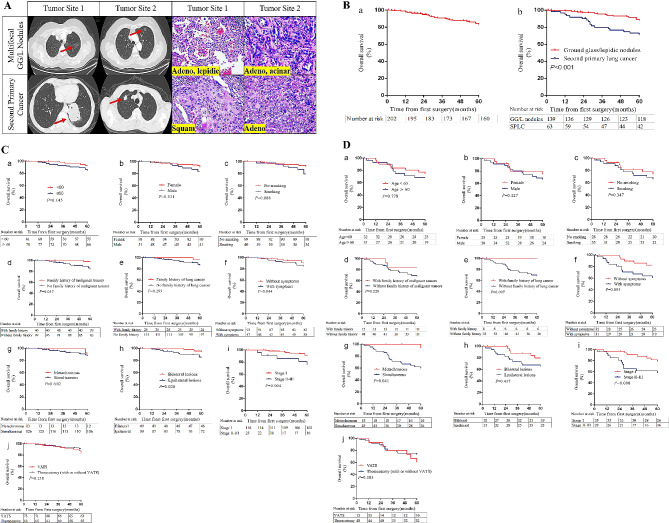
**A** Typical radiologic and pathologic features of different kinds of MPLC. **B-a** Overall survival of MPLC. **B-b** Comparison of overall survival between multiple ground glass nodules and second primary lung cancer. **C** Comparison of overall survival between different groups in multiple ground glass nodule patients. **D** Comparation of overall survival between different groups in second primary lung cancer patients

The data of 5047 consecutive lung cancer patients who underwent surgery in a single center between 2011 and 2013 were reviewed, and 258 (5.1%) patients with multiple lung cancers were identified. According to the 8th edition of the lung cancer staging system, in our cohort, multiple lung cancers were divided into three types: multifocal GG/L nodules, SPLC, and separate tumor nodules. The diagnostic criteria for the three types of multifocal lung cancer were as follows: for multifocal GG/L nodules, the imaging feature was multiple ground glass or part-solid nodules, and the pathologic features were adenocarcinomas with prominent lepidic components (typically varying degrees of AIS, MIA, LPA); for SPLC, the imaging features were two or more distinct masses with imaging characteristics of lung cancer (e.g., spiculated), and the pathological features were different histotypes or different morphological features based on comprehensive histological assessment; for separate tumor nodules, the imaging features were typical lung cancer (e.g., solid, spiculated) with separate solid nodules, and the pathological features were distinct masses with the same morphologic features on comprehensive histological assessment [12]. The flow chart of patient selection is shown in Fig. 1. From January 1, 2011, to December 31, 2013, a total of 258 patients with multifocal lung cancer underwent surgical treatment. Among them, 206 cases were synchronous lung cancers, and 52 cases were metachronous lung cancers. Among the 206 cases of synchronous lung cancers, 126 cases presented with multifocal GG/L nodules, 45 cases were classified as SPLC, 9 cases were separate tumor nodules, and 26 cases were multifocal lung cancers with relationships not sure. Among the 52 cases of metachronous lung cancers, 13 cases exhibited multifocal GG/L nodules, 18 cases were SPLC, and 21 cases were multifocal lung cancers with relationships not sure. Among the selected 258 patients, 139 (53.9%) had multifocal GG/L nodules (adenocarcinoma), and 63 (24.4%) had SPLC. The above two categories of multifocal lung cancers were classified as MPLC (*n* = 202, Fig. 2-A), and 171 were sMPLC, while 31 were mMPLC. Among the 258 patients, 9 (3.5%) had separate tumor nodules, 47 (18.2%) had multiple lung non-adenocarcinomas, and the pathological types of different tumors were the same (e.g., squamous cell carcinoma plus squamous cell carcinoma or small cell carcinoma plus small cell carcinoma), and the relationship between these lesions could not be accurately determined. If multiple lung lesions were diagnosed on chest CT simultaneously, they were diagnosed as simultaneous multiple lung cancers. If the second tumor appeared at a different time (e.g., when the first lesion was diagnosed, no other tumors were clearly observed on chest CT), we diagnosed the patient as metachronous multiple lung cancers (this judgment was interpreted by radiologists). The clinicopathological features of all patients are shown in Table 1. In the following analysis, this study focused on 202 MPLC patients diagnosed according to the 8th lung cancer staging system. Fully informed written consent was obtained from all involved patients, and this study was approved by the medical ethics committee (23/406–4149).

**Table 1:** Clinical features of the 258 patients with multiple lung cancers

Patient characteristics		Results
Age (y), mean ± SD		60.0 ± 8.0
BMI, mean ± SD		24.9 ± 6.1
Male, n (%)		139 (53.9%)
Smoker, n (%)		121 (46.9%)
Drinking history, n (%)		62 (24.0%)
Personal history of neoplasia, n (%)		24 (9.3%)
Family history of neoplasia, n (%)		64 (24.8%)
Family history of lung cancer, n (%)		39 (15.1%)
Past medical history, n (%)		89 (34.5%)
Presented symptoms before the first surgery, n (%)		106 (41.1%)
Prime symptoms before the first surgery, n (%)		
	Cough	66 (25.6%)
	Expectoration	40 (15.5%)
	Bloody sputum	20 (7.8%)
	Chest pain	14 (5.4%)
	Fever	7 (2.7%)
	Hemoptysis	4 (1.6%)
Number of resected tumors, n (%)		
	2	208 (80.6%)
	3	37 (14.3%)
	4	10 (3.9%)
	5	2 (0.8%)
	6	1 (0.4%)
Times the patient underwent surgery, n (%)		
	1	136 (52.7%)
	2	121 (46.9%)
	3	1 (0.4%)
Types of multiple cancers (simultaneous/metachronous)		
	Simultaneous lung cancers	206 (79.8%)
	Metachronous lung cancers	52 (20.2%)
Types of surgery (thoracotomy/VATS)		
	VATS	100 (38.8%)
	Thoracotomy	120 (46.5%)
	Thoracotomy and VATS	38 (14.7%)
Type(s) of resection of multiple lesions		
	Lobectomy + sublobar resection	127 (49.2%)
	Lobectomy + lobectomy	54 (20.9%)
	Sublobar resection + sublobar resection	36 (14.0%)
	Lobectomy	31 (12.0%)
	Pneumonectomy	10 (3.9%)
Relationship of the locations of multiple lesions		
	Ipsilateral tumors	159 (61.6%)
	Bilateral tumors	99 (38.4%)
Pathological type		
	Adenocarcinoma	183 (70.9%)
	Squamous cell carcinoma	37 (14.3%)
	Adenocarcinoma and squamous cell carcinoma	14 (5.4%)
	Adenosine carcinoma and adenocarcinoma	11 (4.3%)
	Adenocarcinoma and large cell carcinoma	3 (1.2%)
	Adenocarcinoma and sarcomatoid carcinoma	2 (0.8%)
	Small cell carcinoma	2 (0.8%)
	Squamous cell carcinoma and large cell carcinoma	1 (0.4%)
	Squamous cell carcinoma and pleomorphic carcinoma	1 (0.4%)
	Squamous cell carcinoma and carcinoid	1 (0.4%)
	Squamous cell carcinoma and small cell carcinoma	1 (0.4%)
	Adenocarcinoma and carcinoid	1 (0.4%)
	Adenocarcinoma and inflammatory myofibroblastoma	1 (0.4%)
Location of lesions		
	Right upper lobe	187 (32.1%)
	Left upper lobe	126 (21.6%)
	Right inferior lobe	111 (19.0%)
	Left inferior lobe	89 (15.3%)
	Right middle lobe	70 (12.0%)
Types of multiple cancers (according to 8th TNM stage)		
	Multifocal GG/L nodules	139 (53.9%)
	Second primary lung cancer	63 (24.4%)
	Multifocal lung cancers with relationship not sure	47 (18.2%)
	Separate tumor nodule	9 (3.5%)
With lung nodules not resected^*^		
	Yes	42 (16.3%)
	No	216 (83.7%)
TNM stage		
	I	151 (58.5%)
	II	23 (89.1%)
	III	37 (14.3%)
	Not sure	47 (18.2%)

^*^With lung nodules not resected: the number of patients with unresected nodules in the lungs

### Clinical information and follow-up

The clinical information of all patients was extracted from the electronic medical record system. The lung cancer-specific overall survival (OS) time of patients was defined as the time from the first operation to the time of death due to lung cancer. Patients were recommended to undergo a postoperative examination every 3 months for 2 years after surgery, every 6 months in years 2–5, and every year after 5 years of follow-up.

### Statistical analysis

Categorical variables are presented as a number and percentage and were analyzed using the Chi-square test or Fisher’s exact test. Continuous variables are presented as the mean and standard deviation and were compared among different groups using the t test. Survival curves were estimated via the Kaplan–Meier method and compared using a log-rank test. Potential factors affecting survival were explored using Cox regression model analysis. All statistical tests were two-sided, and p values of less than 0.05 were considered statistically significant. Statistical analyses were performed using SPSS version (IBM Corp, Armonk, NY) and GraphPad Prism (Version 6.01, GraphPad Software).

## Results

### Comparison of clinicopathological characteristics of different categories of MPLC

#### Comparison of multifocal GG/L nodules and SPLC

We compared the clinicopathological features of 139 multifocal GG/L nodule patients and 63 SPLC patients (Table 2). The results showed that compared with SPLC patients, patients with multifocal GG/L nodules were more likely to be women and nonsmokers, to have more sMPLCs and early stage tumors, to have more unresected nodules, to be more likely to undergo VATS and sublobectomy surgery, to be asymptomatic and to undergo a one-time operation (*P* < 0.05).

**Table 2:** Comparison of multifocal GG/L nodules and second primary lung cancer

Patient characteristics		Multifocal GG/L nodules (*n* = 139)	Second primary lung cancer (*n* = 63)	P value**
Age				0.361
	< 60	61 (43.9%)	32 (50.8%)	
	≥ 60	78 (56.1%)	31 (49.2%)	
BMI				0.114
	≤ 24.9	87 (62.6%)	32 (50.8%)	
	> 24.9	52 (37.4%)	31 (49.2%)	
Sex				**0.002**
	Male	51 (36.7%)	38 (60.3%)	
	Female	88 (63.3%)	25 (39.7%)	
Smoking history				**0.000**
	Yes	40 (28.8%)	35 (55.6%)	
	No	99 (71.2%)	28 (44.4%)	
Drinking history				0.404
	Yes	24 (17.3%)	14 (22.2%)	
	No	115 (82.7%)	49 (77.8%)	
Personal history of neoplasia				0.370
	Yes	12 (8.6%)	8 (12.7%)	
	No	127 (91.4%)	55 (87.3%)	
Family history of neoplasia				0.462
	Yes	40 (28.8%)	15 (23.8%)	
	No	99 (71.2%)	48 (76.2%)	
Family history of lung cancer				0.346
	Yes	25 (18.0%)	8 (12.7%)	
	No	114 (82.0%)	55 (87.3%)	
Past medical history				0.785
	Yes	48 (34.5%)	23 (36.5%)	
	No	91 (65.5%)	40 (63.5%)	
Presenting symptoms before the first surgery				**0.022**
	Yes	47 (33.8%)	32 (50.8%)	
	No	92 (66.2%)	31 (49.2%)	
Number of resected tumors, n (%)				0.224
	2	104 (74.8%)	56 (88.9%)	
	3	26 (18.7%)	6 (9.5%)	
	4	6 (4.3%)	1 (1.6%)	
	5	2 (1.4%)	0 (0.0%)	
	6	1 (0.7%)	0 (0.0%)	
Times the patient underwent surgery, n (%)				**0.043**
	1	83 (59.7%)	28 (44.4%)	
	2	56 (40.3%)	35 (55.6%)	
Types of multiple cancers (simultaneous/metachronous)				**0.000**
	Simultaneous lung cancers	129 (92.8%)	46 (73.0%)	
	Metachronous lung cancers	10 (7.2%)	17 (27.0%)	
Types of surgery (thoracotomy/VATS)				**0.000**
	VATS	73 (52.5%)	15 (23.8%)	
	Thoracotomy	52 (37.4%)	32 (50.8%)	
	Thoracotomy and VATS	14 (10.1%)	16 (25.4%)	
Type(s) of resection of multiple lesions				**0.023**
	Lobectomy + sublobar resection	75 (54.0%)	29 (46.0%)	
	Lobectomy + lobectomy	21 (15.1%)	17 (27.0%)	
	Sublobar resection + sublobar resection	28 (20.1%)	7 (11.1%)	
	Lobectomy	14 (10.1%)	6 (9.5%)	
	Pneumonectomy	1 (0.7%)	4 (6.3%)	
Relationship of the locations of multiple lesions				0.213
	Ipsilateral tumors	90 (64.7%)	35 (55.6%)	
	Bilateral tumors	49 (35.3%)	28 (44.4%)	
With lung nodules not resected^*^				**0.014**
	Yes	34 (24.5%)	6 (9.5%)	
	No	105 (75.5%)	57 (90.5%)	
The highest stage tumor				**0.000**
	I	116 (83.5%)	35 (55.6%)	
	II	7 (5.0%)	12 (19.0%)	
	III	16 (11.5%)	16 (25.4%)	

^*^With lung nodules not resected: the number of patients with unresected nodules in the lungs. **P values of less than 0.05 were considered statistically significant.

#### Comparison of sMPLC and mMPLC

There were 171 sMPLC and 31 mMPLC patients included in this study. The clinicopathological features of the two groups were compared; the results showed that compared with the mMPLC group, the sMPLC group had a higher incidence in women, nonsmokers and nondrinkers, more GG/L type tumors, more ipsilateral tumors, lower patient BMI, more people without a personal malignant tumor history, and more people who underwent VATS and sublobectomy surgery (Table 3). We also compared the second primary lung adenocarcinoma and second primary lung cancers with different pathological types, and the results showed that there were more patients with a smoking history in the second primary lung non-adenocarcinoma group, and BMI was lower in the former group than in the latter group (Supplementary Table 1).

**Table 3:** Comparison of simultaneous and metachronous multiple primary lung cancers

Patient characteristics		Simultaneous multiple primary lung cancers (*n* = 171)	Metachronous multiple primary lung cancers (*n* = 31)	P value**
Age				0.064
	< 60	74 (43.3%)	19 (61.3%)	
	≥ 60	97 (56.7%)	12 (38.7%)	
BMI				**0.013**
	≤ 24.9	107 (62.6%)	12 (38.7%)	
	> 24.9	64 (37.4%)	19 (61.3%)	
Sex				**0.013**
	Male	69 (40.4%)	20 (64.5%)	
	Female	102 (59.6%)	11 (35.5%)	
Smoking history				**0.027**
	Yes	58 (33.9%)	17 (54.8%)	
	No	113 (66.1%)	14 (45.2%)	
Drinking history				**0.037**
	Yes	28 (16.4%)	10 (32.3%)	
	No	143 (83.6%)	21 (67.7%)	
Personal history of neoplasia				**0.048**
	Yes	20 (11.7%)	0 (0.0%)	
	No	151 (88.3%)	31 (100.0%)	
Family history of neoplasia				0.847
	Yes	47 (27.5%)	8 (25.8%)	
	No	124 (72.5%)	23 (74.2%)	
Family history of lung cancer				0.574
	Yes	29 (17.0%)	4 (12.9%)	
	No	142 (83.0%)	27 (87.1%)	
Past medical history				0.966
	Yes	60 (35.1%)	11 (35.5%)	
	No	111 (64.9%)	20 (64.5%)	
Presented symptoms before the first surgery				0.211
	Yes	70 (40.9%)	9 (29.0%)	
	No	101 (59.1%)	22 (71.0%)	
Number of resected tumors, n (%)				0.751
	2	134 (78.4%)	26 (83.9%)	
	3	27 (15.8%)	5 (16.1%)	
	4	7 (4.1%)	0 (0.0%)	
	5	2 (1.2%)	0 (0.0%)	
	6	1 (0.6%)	0 (0.0%)	
Types of multiple cancers (GG/L nodules or second primary lung cancer)				**0.000**
	Multifocal GG/L nodules	126 (73.7%)	13 (41.9%)	
	Second primary lung cancer	45 (26.3%)	18 (58.1%)	
Type(s) of surgery (thoracotomy/ VATS)				**0.000**
	VATS	86 (50.3%)	2 (6.5%)	
	Thoracotomy	69 (40.4%)	15 (48.4%)	
	Thoracotomy and VATS	16 (9.4%)	14 (45.2%)	
Type(s) of resection of multiple lesions				**0.046**
	Lobectomy + sublobar resection	84 (49.1%)	20 (64.5%)	
	Lobectomy + lobectomy	31 (18.1%)	7 (22.6%)	
	Sublobar resection + sublobar resection	34 (19.9%)	1 (3.2%)	
	Lobectomy	19 (11.1%)	1 (3.2%)	
	Pneumonectomy	3 (1.8%)	2 (6.5%)	
Relationship of the locations of multiple lesions				**0.037**
	Ipsilateral tumors	111 (64.9%)	14 (45.2%)	
	Bilateral tumors	60 (35.1%)	17 (54.8%)	
With lung nodules not resected^*^				0.147
	Yes	37 (21.6%)	3 (9.7%)	
	No	134 (78.4%)	28 (90.3%)	
The highest stage tumor				0.499
	I	127 (74.3%)	24 (77.4%)	
	II	15 (8.8%)	4 (12.9%)	
	III	29 (17.0%)	3 (9.7%)	

^*^With lung nodules not resected: the number of patients with unresected nodules in the lungs. **P values of less than 0.05 were considered statistically significant.

### Prognosis and prognostic factors of different disease categories of MPLC

The 1-, 3-, and 5-year OS rates of 202 MPLC patients were 97.98%, 90.18%, and 82.81%, respectively. Patients with GG/L nodules had significantly better survival than was found in SPLC patients (1-, 3-, and 5-year OS, *P* < 0.05) (Fig. 2-B).

#### Prognosis and prognostic factors of GG/L lung cancer and SPLC

The 1-, 3-, and 5-year OS rates of 139 GG/L lung cancer patients were 99.27%, 94.76%, and 87.94%, respectively. Univariate analysis suggested that the presence of symptoms, the tumor location relationship (bilateral or ipsilateral), and the highest tumor stage were prognostic factors. Multivariate analysis suggested that the highest tumor stage was an independent prognostic factor. OS was significantly better in stage I patients (*n* = 116) than in stage II-III patients (*n* = 23) (5-year survival, 90.2% vs. 75.8%, *P* = 0.051). (Supplementary Table 2, Fig. 2-C). The 1-, 3-, and 5-year survival rates of 63 SPLC patients were 95.11%, 78.20%, and 71.29%, respectively. Univariate analysis suggested that the type of MPLC (sMPLC or mMPLC) and the highest tumor stage were prognostic factors, while multivariate analysis demonstrated that the highest tumor stage was an independent prognostic factor. The prognosis was better in stage I patients (*n* = 35) than in stage II-III patients (*n* = 28) (5-year survival, 78.5% vs. 58.0%, *P* = 0.008) (Supplementary Table 3, Fig. 2-D).

#### Prognosis and prognostic factors of sMPLC and mMPLC

The 1-, 3-, and 5-year OS rates of 171 sMPLC patients were 97.60%, 88.34%, and 80.88%, respectively. Univariate analysis suggested that sex, smoking history, family history of cancer, symptoms, category of MPLC (GG/L nodules or SPLC), and the highest tumor stage were prognostic factors for OS (*P* < 0.05). Multivariate analysis demonstrated that sex (*P* = 0.003) and the highest tumor stage (*P* < 0.001) were independent prognostic factors for OS. The prognosis was significantly better in female patients (*n* = 102) than in male patients (*n* = 69) (5-year survival, 88.0% vs. 69.5%, *P* = 0.003). The prognosis was significantly better in stage 1 (*n* = 127) (the highest tumor stage) patients than in stage II-III patients (*n* = 44) (5-year survival, 86.9% vs. 66.2%, *P* < 0.001) (Table 4 and Supplementary Fig. 1). The 1-, 3-, and 5-year OS rates of 31 mMPLC patients were 100%, 100%, and 93.10%, respectively. Univariate analysis identified no significant prognostic factors for mMPLC patients. Although the difference in prognosis between stage I (*n* = 24) patients and stage II-III (*n* = 7) patients was not significant, the prognosis was better in stage I patients than in stage II-III patients (5-year survival, 89.2% vs. 32.1%, *P* = 0.051) (Supplementary Table 4).

**Table 4:** Prognostic factors of simultaneous multiple primary lung cancers

Variable		Univariate cox regression analysis			Multivariate cox regression analysis	
		N (%)	HR (95% CI)	P value**	HR (95% CI)	P value**
Age (years)				0.325		
	< 60	74 (43.3%)	Ref.	Ref.		
	≥ 60	97 (56.7%)	1.404 (0.721–2.688)			
Sex				**0.003**		**0.038**
	Female	102 (59.6%)	Ref.	Ref.	Ref.	
	Male	69 (40.4%)	2.686 (1.447–5.603)		3.115 (1.063–9.126)	
Smoking history				**0.006**		0.639
	Yes	58 (33.9%)	Ref.	Ref.		
	No	113 (66.1%)	0.412 (0.184–0.751)			
Family history of neoplasia				**0.021**		
	Yes	47 (27.5%)	Ref.	Ref.		
	No	124 (72.5%)	3.171 (1.148–4.889)			
Family history of lung cancer				**0.050**		
	Yes	29 (17.0%)	Ref.	Ref.		
	No	142 (83.0%)	3.742 (1.004–5.490)			
Presented symptoms before the first surgery				**0.003**		
	No	101 (59.1%)	Ref.	Ref.		
	Yes	70 (40.9%)	0.378 (0.193–0.739)			
Types of multiple cancers (GG/L nodules or second primary lung cancer)				**< 0.001**		0.066
	Second primary lung cancer	45 (26.3%)	Ref.	Ref.	Ref.	
	Multifocal GG/L nodules	126 (73.7%)	0.278 (0.088–0.407)		0.308 (0.088–1.083)	
Relationship of locations of multiple lesions				1.736		
	Bilateral tumors	60 (35.1%)	Ref.	Ref.		
	Ipsilateral tumors	111 (64.9%)	1.736 (0.825–3.649)			
The highest stage tumor				**< 0.001**		**0.007**
	I	127 (74.3%)	Ref.	Ref.	Ref.	
	II-III	44 (25.7%)	6.127 (2.723–13.790)		2.695 (1.317–5.517)	
Type(s) of surgery (thoracotomy/ VATS)				0.070		
	VATS only	86 (50.3%)	Ref.	Ref.		
	Thoracotomy (with or without VATS)	85 (49.7%)	1.842 (0.952–3.562)			

**P values of less than 0.05 were considered statistically significant

## Discussion

This study systematically describes the landscape of clinicopathological characteristics and prognoses of different disease entities of MPLC. The results show that different categories of MPLC have different characteristics and outcomes. It seems that multiple GG/L nodules and single ground glass nodule patients have similar clinical characteristics; the nonsmokers in our study accounted for 62.9% and women accounted for 55.9% of the 202 MPLC patients [11]. It is commonly thought that multiple adenocarcinomas with ground glass components are more likely to be diagnosed as sMPLC [12]. However, in our study, although most patients with multiple GG/L nodules had sMPLC, a substantial proportion of mMPLC patients (41.9%) had multiple GG/L nodules. It is well known that most lung cancers with ground glass components are in situ or microinvasive adenocarcinomas, and it is therefore rational to assume that patients with multiple GG/L nodules will usually be in an early stage. Our study suggests that multiple GG/L nodules have unique clinical characteristics, and some other studies found that this kind of tumor also have independent molecular characteristics, and the most common genetic mutations are *EGFR*, *ERBB2*, *TP53*, *BRAF*, *RBM10*, and *KRAS* [13, 14]. . These results may support the notion that multiple GG/L nodules should be viewed as an independent disease.

In our study, we also compared the clinicopathological characteristics of patients with second primary lung adenocarcinoma and second primary lung cancers with different pathological types. We found that there were more smokers in the group of second primary lung cancers with different pathological types. Because second primary lung cancers with different pathological types include squamous cell carcinoma and neuroendocrine carcinoma, and there is solid evidence indicating that smoking is closely related to squamous cell and neuroendocrine carcinoma of the lung, this result is reasonable [15].

When comparing the similarities and differences between sMPLC and mMPLC. We found that the majority of sMPLC were GG/L nodules, and these patients therefore showed characteristics similar to those seen in ground glass lung adenocarcinoma patients. Because all of the patients included in this study underwent surgery and had tumors that were diagnosed pathologically, the mMPLC patients underwent regular examination after the first operation. Additionally, there was usually no recurrence or metastasis of the first tumor at the time of the second operation. Therefore, second primary lung cancer is usually identified at an early stage, and these patients have a good prognosis. This result is similar to that of previous studies [16].

We found that sex and the highest tumor stage were independent prognostic factors of sMPLC. Although the ground glass component was not an independent factor for patients in multivariate analysis, prognoses were better in patients with a ground glass component than in those without (5-year survival, 87.5% vs. 59.7%, *P* = 0.066). Therefore, we speculate that the ground glass component plays an important role in judging the prognosis of sMPLC. The prognostic value of sex in sMPLC has rarely been previously evaluated [17]. To further explore the reason for the better prognosis of female patients, we compared the differences between male and female patients and found that more bilateral cases of sMPLC are more common in males than females(Supplementary Table 5), and these may explain the better prognosis of female sMPLC patients.

The proportion of multiple GG/L nodules was higher in female patients than in male patients, and it is known that multiple GG/L lung cancer has an indolent clinical course; thus, these patients have a better prognosis. The results of this study suggest that regardless of the MPLC category, the highest tumor stage plays a decisive role in predicting prognosis. Therefore, we believe that the prognosis of MPLC mainly depends on the highest stage of the tumor lesions. This conclusion also provides a basis for prioritizing the resection and treatment of the main lung lesions.

Given that this was a retrospective, single-institute study, selection bias and time-trend bias were inevitable. For example, our conclusion is based on results obtained in a Chinese population, and the proportions of never smokers and adenocarcinoma were relatively high. In the future, larger patient cohorts, longer follow-up times, and multicenter data are needed to fully explain the clinical characteristic spectrum and prognostic factors of MPLC to provide more guidance for the accurate diagnosis and rational treatment of patients.

### Electronic supplementary material

Below is the link to the electronic supplementary material.

Supplementary Material 1

Supplementary Material 2
