# Supplementary material for: The characteristics and prognosis of different disease patterns of multiple primary lung cancers categorized according to the 8th edition lung cancer staging system
Source: J Cardiothorac Surg. 2024 Apr 10;19:200. doi: 10.1186/s13019-024-02652-8 (PMC11008024; doi:10.1186/s13019-024-02652-8)
Supplement: Supplementary file 1 — Supplementary Material 1 [file 13019_2024_2652_MOESM1_ESM.docx]

**Supplementary table 1.** **Comparison of second primary lung adenocarcinoma and second primary lung cancers with different pathological types.**

| Patient characteristics | | Second primary lung cancer (only adenocarcinoma) (n=35) | Second primary lung cancer (different pathological types) (n=28) | *P* value |
| --- | --- | --- | --- | --- |
| Age (y), mean ± SD | |  |  | 0.260 |
|  | < 60 | 20 (57.1%) | 12 (42.9%) |  |
|  | ≥ 60 | 15 (42.9%) | 16 (57.1%) |  |
| BMI | |  |  | **0.015** |
|  | ≤ 24.9 | 13 (37.1%) | 19 (67.9%) |  |
|  | > 24.9 | 22 (62.9%) | 9 (32.1%) |  |
| Sex | |  |  | 0.107 |
|  | Male | 18 (51.4%) | 20 (71.4%) |  |
|  | Female | 17 (48.6%) | 8 (28.6%) |  |
| Smoking history | |  |  | **0.005** |
|  | Yes | 14 (40.0%) | 21 (75.0%) |  |
|  | No | 21 (60.0%) | 7 (25.0%) |  |
| Drinking history | |  |  | 0.090 |
|  | Yes | 5 (14.3%) | 9 (32.1%) |  |
|  | No | 30 (85.7%) | 19 (67.9%) |  |
| Personal history of neoplasia | |  |  | 0.236 |
|  | Yes | 6 (17.1%) | 2 (7.1%) |  |
|  | No | 29 (82.9%) | 26 (92.9%) |  |
| Family history of neoplasia | |  |  | 0.112 |
|  | Yes | 11 (31.4%) | 4 (14.3%) |  |
|  | No | 24 (68.6%) | 24 (85.7%) |  |
| Family history of lung cancer | | |  | 0.236 |
|  | Yes | 6 (17.1%) | 2 (7.1%) |  |
|  | No | 29 (82.9%) | 26 (92.9%) |  |
| Presented symptoms before the first surgery | | |  | 0.159 |
|  | Yes | 15 (42.9%) | 17 (68.0%) |  |
|  | No | 20 (57.1%) | 11 (39.3%) |  |
| Number of resected tumors, n (%) | |  |  | 0.501 |
|  | 2 | 32 (91.4%) | 24 (85.7%) |  |
|  | 3 | 3 (8.6%) | 3 (10.7%) |  |
|  | 4 | 0 (0.0%) | 1 (3.6%) |  |
| Types of multiple cancers (Simultaneous/metachronous) | | |  | 0.800 |
|  | Simultaneous lung cancers | 26 (74.3%) | 20 (71.4%) |  |
|  | Metachronous lung cancers | 9 (25.7%) | 8 (28.6%) |  |
| Type(s) of surgery (thoracotomy/ VATS) | | |  | 0.436 |
|  | VATS | 10 (28.6%) | 5 (17.9%) |  |
|  | Thoracotomy | 18 (51.4%) | 14 (50.0%) |  |
|  | Thoracotomy and VATS | 7 (20.0%) | 9 (32.1%) |  |
| Type(s) of resection of multiple lesions | | |  | 0.354 |
|  | Lobectomy + sublobar resection | 19 (54.3%) | 10 (35.7%) |  |
|  | Lobectomy+lobectomy | 10 (28.6%) | 7 (25.0%) |  |
|  | Sublobar resection+ sublobar resection | 3 (8.6%) | 4 (14.3%) |  |
|  | Lobectomy | 2 (5.7%) | 4 (14.3%) |  |
|  | Pneumonectomy | 1 (2.9%) | 3 (10.7%) |  |
| Relationship of the locations of multiple lesions | | |  | 0.192 |
|  | Ipsilateral tumors | 22 (62.9%) | 13 (46.4%) |  |
|  | Bilateral tumors | 13 (37.1%) | 15 (53.6%) |  |

**Supplementary table 2. Prognostic factors of multifocal GG/L nodules**

| Variable | | Univariate cox regression analysis | | | Multivariate cox regression analysis | |
| --- | --- | --- | --- | --- | --- | --- |
|  |  | N (%) | HR (95% CI) | P value | HR (95% CI) | P value |
| Age (years) | |  |  | 0.145 |  |  |
|  | < 60 | 61 (43.9%) | Ref. | Ref. |  |  |
|  | ≥ 60 | 78 (56.1%) | 2.000 (0.803-4.856) |  |  |  |
| Sex | |  |  | 0.331 |  |  |
|  | Female | 88 (63.3%) | Ref. | Ref. |  |  |
|  | Male | 51 (36.7%) | 1.551 (0.628-4.059) |  |  |  |
| Smoking history | |  |  | 0.088 |  |  |
|  | No | 99 (71.2%) | Ref. | Ref. |  |  |
|  | Yes | 40 (28.8%) | 2.140 (0.884-6.337) |  |  |  |
| Family history of neoplasia | |  |  | 0.057 |  |  |
|  | No | 99 (71.2%) | Ref. | Ref. |  |  |
|  | Yes | 40 (28.8%) | 0.267 (0.145-1.024) |  |  |  |
| Family history of lung cancer | |  |  | 0.293 |  |  |
|  | Yes | 25 (18.0%) | Ref. | Ref. |  |  |
|  | No | 114 (82.0%) | 2.148 (0.596-5.602) |  |  |  |
| Presented symptoms before the first surgery | |  |  | **0.044** |  | 0.167 |
|  | Yes | 47 (33.8%) | Ref. | Ref. | Ref. |  |
|  | No | 92 (66.2%) | 0.410 (0.140-0.971) |  | 0.516 (0.201-1.321) |  |
| Types of multiple cancers (Simultaneous/metachronous) | | | | 0.650 |  |  |
|  | Simultaneous multiple primary lung cancers | 126 (90.6%) | Ref. | Ref. |  |  |
|  | Metachronous multiple primary lung cancers | 13 (93.5%) | 0.726 (0.183-2.888) |  |  |  |
| Relationship of locations of multiple lesions | | |  | **0.026** |  | 0.258 |
|  | Bilateral tumors | 49 (35.3%) | Ref. | Ref. | Ref. |  |
|  | Ipsilateral tumors | 90 (64.7%) | 2.867 (1.134-7.249) |  | 1.905 (0.624-5.819) |  |
| The highest stage tumor | |  |  | **0.004** |  | **0.029** |
|  | I | 116 (83.5%) | Ref. | Ref. | Ref. |  |
|  | II-III | 23 (16.5%) | 6.898 (1.872-25.42) |  | 2.977 (1.117-7.932) |  |
| Type(s) of surgery (thoracotomy/ VATS) | | |  | 0.238 |  |  |
|  | VATS only | 73 (52.5%) | Ref. | Ref. |  |  |
|  | Thoracotomy (with or without VATS) | 66 (47.5%) | 1.731 (0.696-4.308) |  |  |  |

**Supplementary table 3. Prognostic factors of second primary lung cancer**

| Variable | | Univariate cox regression analysis | | | Multivariate cox regression analysis | |
| --- | --- | --- | --- | --- | --- | --- |
|  |  | N (%) | HR (95% CI) | P value | HR (95% CI) | P value |
| Age (years) | |  |  | 0.778 |  |  |
|  | < 60 | 32 (50.8%) | Ref. | Ref. |  |  |
|  | ≥ 60 | 31 (49.2%) | 1.124 (0.497-2.549) |  |  |  |
| Sex | |  |  | 0.127 |  |  |
|  | Female | 25 (39.7%) | Ref. | Ref. |  |  |
|  | Male | 38 (60.3%) | 2.030 (0.833-4.372) |  |  |  |
| Smoking history | |  |  | 0.347 |  |  |
|  | Yes | 35 (55.6%) | Ref. | Ref. |  |  |
|  | No | 28 (44.4%) | 0.673 (0.297-1.526) |  |  |  |
| Family history of neoplasia | |  |  | 0.226 |  |  |
|  | Yes | 15 (23.8%) | Ref. | Ref. |  |  |
|  | No | 48 (76.2%) | 2.080 (0.694-4.731) |  |  |  |
| Family history of lung cancer | |  |  | 0.097 |  |  |
|  | Yes | 8 (12.7%) | Ref. | Ref. |  |  |
|  | No | 55 (87.3%) | 3.132 (0.815-12.040) |  |  |  |
| Presented symptoms before the first surgery | | |  | 0.093 |  |  |
|  | No | 31 (49.2%) | Ref. | Ref. |  |  |
|  | Yes | 32 (50.8%) | 2.022 (0.889-4.600) |  |  |  |
| Type of multiple cancers (Simultaneous/metachronous) | | | | **0.041** |  |  |
|  | Simultaneous | 45 (71.4%) | Ref. | Ref. | Ref. | 0.100 |
|  | Metachronous | 18 (28.6%) | 0.348 (1.056-5.701) |  | 0.398 (0.133-1.194) |  |
| Relationship of locations of multiple lesions | | |  | 0.437 |  |  |
|  | Bilateral tumors | 28 (44.4%) | Ref. | Ref. |  |  |
|  | Ipsilateral tumors | 35 (55.6%) | 1.391 (0.611-3.140) |  |  |  |
| The highest stage tumor | |  |  | **0.008** |  | **0.025** |
|  | I | 35 (55.6%) | Ref. | Ref. | Ref. |  |
|  | II-III | 28 (44.4%) | 3.025 (1.360-7.307) |  | 2.704 (1.135-6.442) |  |
| Type(s) of surgery (thoracotomy/ VATS) | | |  | 0.585 |  |  |
|  | VATS only | 15 (23.8%) | Ref. | Ref. |  |  |
|  | Thoracotomy (with or without VATS) | 48 (76.2%) | 1.287 (0.491-3.589) |  |  |  |

**Supplementary table 4. Prognostic factors of metachronous multiple primary lung cancers**

| Variable |  | Univariate cox regression analysis | | |
| --- | --- | --- | --- | --- |
|  |  | N (%) | HR (95% CI) | P value |
| Age (years) | |  |  | 0.931 |
|  | < 60 | 19 (61.3%) | Ref. | Ref. |
|  | ≥ 60 | 12 (38.7%) | 1.077 (0.201-5.787) |  |
| Sex | |  |  | 0.534 |
|  | Female | 11 (35.5%) | Ref. | Ref. |
|  | Male | 20 (64.5%) | 1.808 (0.280-11.680) |  |
| Smoking history | |  |  | 0.243 |
|  | Yes | 17 (54.8%) | Ref. | Ref. |
|  | No | 14 (45.2%) | 0.348 (0.059-2.047) |  |
| Family history of neoplasia | |  |  | 0.405 |
|  | Yes | 8 (25.8%) | Ref. | Ref. |
|  | No | 23 (74.2%) | 2.095 (0.368-11.930) |  |
| Family history of lung cancer | |  |  | 0.334 |
|  | Yes | 4 (12.9%) | Ref. | Ref. |
|  | No | 27 (87.1%) | 3.181 (0.305-33.210) |  |
| Presented symptoms before the first surgery | |  |  | 0.552 |
|  | No | 9 (29.0%) | Ref. | Ref. |
|  | Yes | 22 (71.0%) | 1.655 (0.315-8.704) |  |
| Types of multiple cancers (GG/L nodules or second primary lung cancer) | | | | 0.533 |
|  | Second primary lung cancer | 18 (58.1%) | Ref. | Ref. |
|  | Multifocal GG/L nodules | 13 (41.9%) | 0.599 (0.120-2.993) |  |
| Relationship of locations of multiple lesions | | |  | 0.523 |
|  | Bilateral tumors | 17 (54.8%) | Ref. | Ref. |
|  | Ipsilateral tumors | 14 (45.2%) | 1.688 (0.339-8.414) |  |
| The highest stage tumor | |  |  | 0.051 |
|  | I | 24 (77.4%) | Ref. | Ref. |
|  | II-III | 7 (22.6%) | 5.840 (0.994-34.310) |  |

**Supplementary table 5. Comparison of female and male sMPLC patients.**

| Patient characteristics | | Female patients (n=102) | Male patients(n=69) | *P* value |
| --- | --- | --- | --- | --- |
| Age (years) | |  |  | 0.965 |
|  | < 60 | 44 | 30 |  |
|  | ≥ 60 | 58 | 39 |  |
| BMI | |  |  | 0.955 |
|  | ≤ 24.9 | 64 | 43 |  |
|  | > 24.9 | 38 | 26 |  |
| Smoking history | |  |  | **0.000** |
|  | Yes | 5 | 53 |  |
|  | No | 97 | 16 |  |
| Drinking history | |  |  | **0.000** |
|  | Yes | 1 | 27 |  |
|  | No | 101 | 42 |  |
| Personal history of neoplasia | |  |  | 0.652 |
|  | Yes | 11 | 9 |  |
|  | No | 91 | 60 |  |
| Family history of neoplasia | |  |  | **0.037** |
|  | Yes | 34 | 13 |  |
|  | No | 68 | 56 |  |
| Family history of lung cancer | |  |  | 0.480 |
|  | Yes | 19 | 10 |  |
|  | No | 83 | 59 |  |
| Past medical history | |  |  | **0.027** |
|  | Yes | 29 | 31 |  |
|  | No | 73 | 38 |  |
| Presented symptoms before the first surgery | | |  | 0.383 |
|  | Yes | 39 | 31 |  |
|  | No | 63 | 38 |  |
| Number of resected tumors, n (%) | |  |  | 0.430 |
|  | 2 | 81 | 53 |  |
|  | 3 | 14 | 13 |  |
|  | 4 | 4 | 3 |  |
|  | 5 | 2 | 0 |  |
|  | 6 | 1 | 0 |  |
| Types of multiple cancers (GG/L nodules or second primary lung cancer) | | | | **0.015** |
|  | Multifocal GG/L nodules | 82 | 44 |  |
|  | Second primary lung cancer | 20 | 25 |  |
| Type(s) of surgery (thoracotomy/ VATS) | |  |  | **0.048** |
|  | VATS | 55 | 31 |  |
|  | Thoracotomy | 42 | 27 |  |
|  | Thoracotomy and VATS | 5 | 11 |  |
| Type(s) of resection of multiple lesions | |  |  | 0.814 |
|  | Lobectomy + sublobar resection | 51 | 33 |  |
|  | Lobectomy + lobectomy | 17 | 14 |  |
|  | Sublobar resection+ sublobar resection | 22 | 12 |  |
|  | Lobectomy | 11 | 8 |  |
|  | Pneumonectomy | 1 | 2 |  |
| Relationship of locations of multiple lesions | |  |  | **0.027** |
|  | Ipsilateral tumors | 73 | 38 |  |
|  | Bilateral tumors | 29 | 31 |  |
| With lung nodules not resected | |  |  | 0.465 |
|  | Yes | 24 | 13 |  |
|  | No | 78 | 56 |  |
| The highest stage tumor | |  |  | 0.956 |
|  | I | 75 | 52 |  |
|  | II | 9 | 6 |  |
|  | III | 18 | 11 |  |
