# Supplementary figures and images for: The characteristics and prognosis of different disease patterns of multiple primary lung cancers categorized according to the 8th edition lung cancer staging system
Source: J Cardiothorac Surg. 2024 Apr 10;19:200. doi: 10.1186/s13019-024-02652-8 (PMC11008024; doi:10.1186/s13019-024-02652-8)

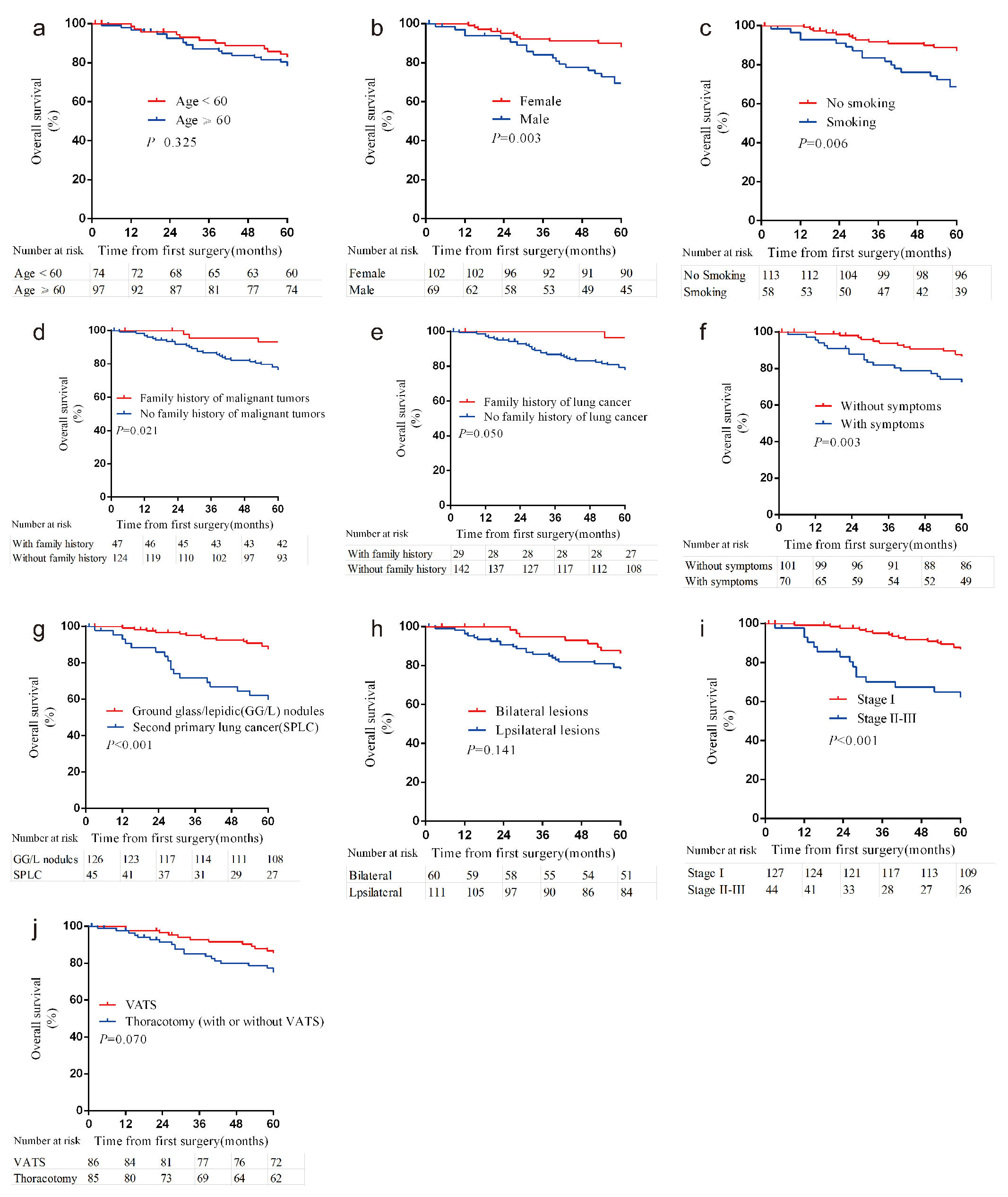

Supplement: Supplementary file 2 — Supplementary Material 2 [file 13019_2024_2652_MOESM2_ESM.jpg]
